# Acknowledgement to Reviewers of *Diagnostics* in 2017

**DOI:** 10.3390/diagnostics8010006

**Published:** 2018-01-10

**Authors:** Diagnostics Editorial Office

**Affiliations:** MDPI AG, St. Alban-Anlage 66, 4052 Basel, Switzerland

Peer review is an essential part in the publication process, ensuring that *Diagnostics* maintains high quality standards for its published papers. In 2017, a total of 57 papers were published in the journal. Thanks to the cooperation of our reviewers, the median time to first decision was 25 days and the median time to publication was 61 days. The editors would like to express their sincere gratitude to the following reviewers for their time and dedication in 2017:
Adhikari, PrakashMayo, RayAkif, TurnaMiederer, MatthiasAlmerich-Silla, José ManuelMilano, FrancescoAmarasena, NajithMiles, AnneAndreoni, GiuseppeMirza, Aleem K.H.Banitsas, KonstantinosMora-Gutiérrez, José MaríaBankhead, ClareMorbelli, SilviaBaptista, Pedro VianaMuyldermans, SergeBecker, Therese M.Mystkowska, JoannaBiermann, MartinNarod, Steven A.Bisharat, NaielNyström, PärBoisselier, ElodieOćwieja, MagdalenaBowen, Deborah J.Okeke, Nwora LanceBricout, MarineOkumura, NobuakiCai, LishengOrsi, AndreaCarpenter, GuyPark, Mi-SukCatry, BoudewijnParnetti, LucillaCavoretto, PaoloPiersma, DafneCehofski, LassePinsky, Paul F.Chatterjee, JhinukPolanec, StephanChen, Ruei-mingPond, DimityChen, ZhihangPovlsen, SebastianChiu, Ka Fung PeterPrabhulkar, ShradhaCombes, Jean-DamienPriefer, RonnyCoombs, Robert W.Pulford, KarenCraven, Michael P.Ramani, VijayDe Biase, DarioRamaraj, PanduranganDelabaere, AmélieRispoli, JosephDi Bartolomeo, AntonioRitter, KerstinDimitrakopoulou-Strauss, AntoniaRobert, LidiaDorward, JienchiRogerson, Stephen J.Eedunuri, Vijay KumarRostaing, LionelEhrnhoefer, DagmarSachpekidis, ChristosEkpo, Ernest U.Sai, Kiran Kumar SolingapuramElgazzar, Abdelhamid H.Saini, ShikhaFasano, FabrizioSaponara, SergioFerrante, Mark A.Sarkozy, ClémentineFleischer, Arthur C.Sathekge, MikeFrancis, SusanSchachter, Steven C.Franklin, Gary M.Schelhaas, SonjaGiubileo, FilippoSchoder, HeikoGlaser, Gretchen E.Schrum, AdamGordon, Catherine A.Seiler, StephenHambrock, ThomasShea, Christopher M.Han, Seung HeeShieh, Tzong-MingHendrikse, HarrySinger, Eric A.Hingorani, DinaSingh, VirtajHolland, KatharinaSmith, Heather F.Hsieh, KuangwenSoh, Jun HuiIslam, KamrulSpanakis, Emmanouil G.Iwata, JunichiSubhi, YousifJakowski, Joseph D.Sun, MengJeong, Keun-YeongSundar, SudhaJia, YinnongSzubert, SebastianJiménez-Bonilla, Julio FranciscoTakebayashi, ShigeoJin, YanTanaka, Yumiko OishiKanoun, SalimTemkin, SarahKantarci, AlpdoganThompson, TrevorKara, H. VolkanTsai, Fuu-JenKaushik, AjeetUlum, Mokhamad FakhrulKoshiyama, MasafumiUusi-Rasi, KirstiKubina, RobertVanhomwegen, JessicaLa Rocca, Renato V.Vashist, Sandeep KumarLakhal-Chaieb, LajmiVenkatesh, Alagarswamy G.Lamb, Benjamin W.Venturella, RobertaLång, KristinaViggiano, DavideLee, Kwan HyiVougas, KostasLee, ZhenghongWarwick, JaneLeitman, I. MichaelWei, ChangyongLeung, FelixWeiergräber, MarcoLewis, MarkWhelan, Rebecca J.Li, Xiang-GuoWilczek, BrigitteLicitra, LisaWillmann, Jürgen K.Liu, Shing-HongWoods, Christopher WildrickLiu, XiaokeXu, WangLiu, YuYakobson, Boris A.Løbner-Olesen, AndersYeh, Chih-koLópez, Carmen BlancoYou, XiaonaLum, Ying WeiZanello, AlessandroMariño, Inés P.Zhang, MingMarottoli, Richard A.Zhu, QuingMarra, PaoloZhu, ZhaohuiMashamba-Thompson, Tivani P.



